# Adherence to Pharmacotherapies After Heart Transplantation in Relation to Multimorbidity and Socioeconomic Position: A Nationwide Register-Based Study

**DOI:** 10.3389/ti.2023.11676

**Published:** 2023-10-11

**Authors:** Rikke E. Mols, István Bakos, Brian B Løgstrup, Erzsébet Horváth-Puhó, Finn Gustafsson, Hans Eiskjær

**Affiliations:** ^1^ Department of Cardiology, Aarhus University Hospital, Aarhus, Denmark; ^2^ Department of Clinical Medicine, Aarhus University, Aarhus, Denmark; ^3^ Department of Clinical Epidemiology, Aarhus University Hospital and Aarhus University, Aarhus, Denmark; ^4^ Department of Cardiology, University Hospital of Copenhagen, Copenhagen, Denmark; ^5^ Department of Clinical Medicine, University of Copenhagen, Copenhagen, Denmark

**Keywords:** heart transplantation, pharmacological management regime, immunosuppression, socioeconomic position, multimorbidity

## Abstract

No studies have examined the impact of multimorbidity and socioeconomic position (SEP) on adherence to the pharmacological therapies following heart transplantation (HTx). Using nationwide Danish registers, we tested the hypothesis that multimorbidity and SEP affect treatment patterns and adherence to pharmacological therapies in first-time HTx recipients. Pharmacological management included cost-free immunosuppressants and adjuvant medical treatment (preventive and hypertensive pharmacotherapies; loop diuretics). We enrolled 512 recipients. The median (IQR) age was 51 years (38–58 years) and 393 recipients (77%) were males. In recipients with at least two chronic diseases, prevalence of treatment with antihypertensive pharmacotherapies and loop diuretics was higher. The overall prevalence of adherence to treatment with tacrolimus or mycophenolate mofetil was at least 80%. Prevalence of adherence to preventive pharmacotherapies ranged between 65% and 95% and between 66% and 88% for antihypertensive pharmacotherapies and loop diuretics, respectively. In socioeconomically disadvantaged recipients, both the number of recipients treated with and adherence to cost-free everolimus, lipid modifying agents, angiotensin-converting enzyme/angiotensin II inhibitors, calcium channel blockers, and loop diuretics were lower. In recipients with multimorbidity, prevalence of treatment with antihypertensive pharmacotherapies and loop diuretics was higher. Among socioeconomically disadvantaged recipients, both number of patients treated with and adherence to cost-free everolimus and adjuvant pharmacotherapies were lower.

## Graphical Abstract

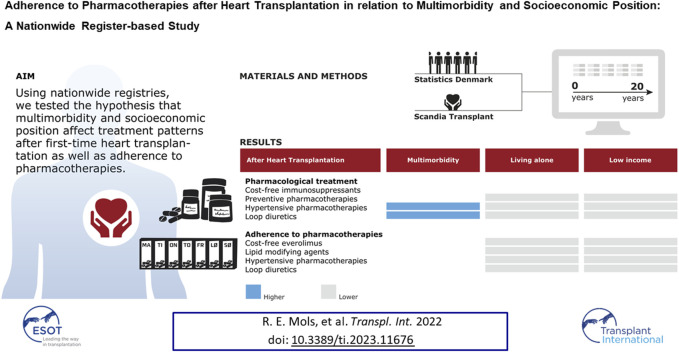

## Introduction

Heart transplantation (HTx) is the ultimate treatment for end-stage heart failure [1, 2]. HTx recipients require life-long pharmacological treatment [2, 3]. Improvements in immunosuppressive and adjuvant medical treatment to avoid graft rejection has improved survival in HTx recipients [4–6]. Thus, pharmacological treatment has become more complex to prevent or treat post-transplant complications and comorbidities [5, 7, 8]. Polypharmacy including up to sixteen pharmacotherapies is seen in one-third of recipients 5 years after HTx [7]. A single-center study suggested that especially regular and accurate intake of immunosuppressants is vital for organ survival [9].

Previous studies have reported sub-optimal self-reported adherence to medical therapies following HTx [10–13]. A cross-sectional study described that the medication complexity score, and the rate of new onset multimorbidity were alarmingly high in Spanish HTx recipients [14]. Addressing long-term non-adherence to medical therapies is crucial to achieve optimal post-HTx outcomes [9, 12, 15].

We previously described the patterns of multimorbidity and socioeconomic position (SEP) concerning the overall pharmacological services utilization after HTx. The study reported a higher number of prescriptions in recipients with three or more comorbidities, and a lower number of prescriptions in recipients within the lowest income group or among those living alone [16]. In the United States and the United Kingdom, lower SEP is documented to be associated with poorer HTx outcome [17–20] and it could be hypothesized that a plausible explanation may be found in a socioeconomic gradient in non-adherence to pharmacotherapies [9, 17]. However, no studies have examined the impact of multimorbidity and SEP on adherence to the pharmacological therapies in post-HTx recipients. Moreover, the majority of earlier studies of adherence to post-transplant pharmacotherapy have utilized self-reported measures of adherence [21] in countries without universal healthcare systems. Using nationwide registries, we tested the hypothesis that multimorbidity and SEP affect treatment patterns after first-time HTx as well as adherence to pharmacotherapies.

## Materials and Methods

### Design and Setting

We conducted a nationwide cohort study in first-time HTx recipients in Denmark between 1 January 1995 and 31 December 2018. Denmark has two HTx centers at University Hospital of Copenhagen and Aarhus University Hospital. The Danish healthcare system is primarily tax-financed with free access to both in-hospital and general practice healthcare services for all Danish citizens. The Danish Civil Registration System (CRS) records vital status using a unique ten-digit identifier assigned to all citizens at birth or immigration. The personal identifier enables access to individual-level data across health and administrative registers [22]. General reimbursement is given for prescription medicine at community pharmacies apart from a minor co-payment, and immunosuppressants are provided free of charge from hospital pharmacies (independent of multimorbidity and SEP) [22]. HTx recipients in this study were followed until 31 December 2018, migration or the date of all-cause mortality, whichever came first.

### Study Cohort and Characteristics

We used the complete Scandiatransplant Database (STD) [23] to construct a cohort of first-time HTx recipients identified by the International Classification of Diseases system Revision (ICD-10 code: DZ94.1). The index date was the date of surgery in the STD. Information regarding recipient age and gender at index date was extracted from the CRS [24].

We identified morbidities from the Danish National Patient Registry (DNPR) [25] and in the Psychiatric Central Research Register (PCRR) [26]. Diagnoses are coded according to ICD-8/10 [25, 26] and somatic and mental morbidities 10 years prior to index date were defined (Supplementary Table S1). To address multimorbidity, we used an algorithm applied in previous Danish studies [16, 27] including a high number of specific physical and mental chronic morbidities, divided into 11 comprehensive chronic disease groups: cardiovascular disease, hypertension, diabetes, obstructive pulmonary disease, cancer, neurological disorder, arthritis, bowel disease, liver disease, kidney disease, and mental illness. This Danish algorithm defined multimorbidity as the co-occurrence of two or more chronic conditions included in the 11 comprehensive chronic disease groups. We summarized the number of chronic diseases, excluding cardiovascular diseases (Supplementary Table S1).

Four different individual-level SEP indicators were applied from Statistic Denmark and CRS: cohabitation status, highest attained educational degree, employment status, and personal income [22, 24]. Information on cohabitation status at index date was defined as living alone or cohabitation. We used the highest attained educational degree in the calendar year before the index date and grouped recipients into four categories: low (no formal education, primary and lower secondary education); medium (upper secondary education and academy profession degree); high (bachelor and above); not completed any education (recipients under age of 16 years). Employment status the year before index date was divided into working, not working, early retirement, state pension, and under education. Based on the annual percentiles in the Danish population, we classified income into percentiles and used the 25th percentile as a cut-off point for low (≤25th percentile) and medium-high (>25th percentile) income (Supplementary Table S2).

### Pharmacological Management Regime

The pharmacological management regime [3] was defined by used treatment with cost-free immunosuppressants such as ciclosporin, tacrolimus, everolimus, mycophenolate mofetil, and prophylactic anti-infectious medication such as valganciclovir, and sulfamethoxazole with trimethoprim. Glucocorticoids were not totally cost-free and were generally tapered down during the first year and stopped after 12–18 months depending on biopsy history. Patients also had to pay a minor part of the costs of adjuvant medical treatment such as preventive pharmacotherapies (antiplatelet agents, lipid lowering agents) as well as antihypertensive pharmacotherapies (ACE/AT [Angiotensin-converting enzyme/angiotensin] II inhibitors, aldosterone antagonists, calcium channel blockers, thiazides) and loop diuretics (furosemide or bumetanide) (Supplementary Table S3). Lipid modifying agents (primarily Pravastatin) was given as a standard to all recipients. In case of statin intolerance, ezetimibe was prescribed. Antiplatelet therapy was not routinely given to all recipients but only on specific indications.

Data on reimbursed pharmacotherapies were provided by the Danish National Prescription Registry (NPR) [28]. Records include Anatomic Therapeutic Chemical (ACT) code, date of reimbursement, strength and formulation, and number of tables reimbursed. However, no information on prescribing indication or prescribed daily dose is available in the DNPR [28]. We defined treatment with medical therapies (ACT code) as one or more reimbursed prescription within 180 days intervals after HTx. The hospital pharmacy at Aarhus University Hospital has electronically recorded use of cost-free immunosuppressants by date of dispensing, strength and formulation, and the number of tablets dispensed. We used this information in a sub-analysis including HTx recipients from Transplant Center Aarhus from 1 January 1995 to 31 December 2018 [16].

Prevalence of medical treatment within the pharmacological management regime was estimated by 180 days intervals during follow-up. We only included HTx recipients with a complete follow-up of a least 365 days and prescriptions redeemed (≥1) in the first and/or second 180 days interval after index date.

Polypharmacy before HTx (baseline) was defined as at least one reimbursed prescription related to ≥5 agents within the Cardiovascular ACT index 180 days prior to index date.

### Adherence to Pharmacotherapies

To describe adherence to used pharmacotherapies, we estimated the proportion of days covered (PDC) [29] within 180 days intervals in recipients treated with medical therapies. The first 180 days after index date were considered as a blanking period to allow breaks, change, or up-titration of medical therapies. We applied 80% of days covered as the threshold for adherence and PDC < 80% as non-adherence [29]. Since data on prescribed daily dose is not available in neither the NPR nor in pharmacy records at Aarhus University Hospital, we calculated the gold standard for prescribed daily dose of immunosuppressants and adjuvant medical treatment by two different methods: a) a fixed dosing regimen or b) an estimated dosing regimen. Based on clinical guidelines [3] and local practice, a fixed daily dose of two tablets or one tablet per day was used for cost-free immunosuppressants. In line with preventive guidelines [2], a fixed daily dose of one tablet per day was chosen as the gold standard in glucocorticoids and preventive pharmacotherapies. In antihypertensives and loop diuretics, we calculated the median daily dose (MDD) by all prescriptions in the period 180–360 days after index date (Supplementary Table S4). This individual MDD-1 was used as the gold standard daily dose during the next five 180 days intervals. Next, a new individual MDD (MDD-2) was estimated using all prescriptions in the period 1,081–1,260 days after index date. The MDD-2 was used as the gold standard daily dose in the period 1,261–2,160 days after index date (Supplementary Figure S1).

In case of a break in reimbursed prescriptions of more than 365 days in HTx recipients, we defined this as a +365 days break if recipients survived or did not emigrate in this period. The HTx recipients were followed to end of pills within this break of 365 days. We allowed a 7 days grace period to account for short discontinuations. As the DNPR and the pharmacy at Aarhus University Hospital do not capture pharmacotherapies dispensed during hospitalizations, HTx recipients were assumed to receive medical therapies in-hospital if readmitted for more than 7 days. If HTx recipients had pills left within the 365 days of follow-up, we pragmatically decided that maximum 90 pills were included in the next follow-up period (several pharmacotherapies have 3 months of durability). This was decided as recipients collect prescriptions lasting longer than the follow-up interval.

### Statistical Analysis

We characterized HTx recipients according to baseline characteristics by presenting median and interquartile range (25th–75th percentile [IQR]) or numbers (n) and percentage (%).

To assess the potential influence of multimorbidity and socioeconomic disadvantage, we also dichotomized educational degree (low education [low] versus medium-high education [medium + high]) and employment status (unemployed [not working, early retirement] versus employed [working, state pension, under education]) (Supplementary Table S2). The categorization was based on general epidemiological assumptions used in Denmark. Recipients with missing data were not included (<0.01%).

Over time, used treatment within the pharmacological management regimes after HTx was examined. First, we graphically displayed prevalence curves for immunosuppressants and adjuvant pharmacotherapies by 180 days intervals during follow in recipients still alive and not emigrated. Next, we described the influence of multimorbidity and SEP on the used pharmacotherapies by graphically depicted prevalence curves stratified by the dichotomized variables of multimorbidity and SEP. Similarly, we followed recipients still alive or not emigrated within 180 days periods. We evaluated the prevalence of non-adherence (PDC < 80%) for used pharmacotherapies by descriptive illustrations. HTx recipients were followed by 180 days intervals until censoring event (+365 days break, mortality, emigration) or end of follow-up. Then, graphical curves were stratified according to the dichotomized variables of multimorbidity and SEP to illustrate the influence of these baseline variables. Sensitivity illustrations were performed as distributed plots for PDC outcomes during follow-up. According to the Danish Data Protection Agency and IRB (Institutional Review Boards) approval, scientists are not allowed to report numbers less than five or aggregated results based on less than five observations. These are thus marked as NA (not available) in the manuscript or ended follow-up in graphical displays. Moreover, if prevalence was less than 20% of pharmacotherapies during 180 days follow-up intervals, adherence outcomes stratified by multimorbidity and socioeconomic disadvantage were not presented.

Analyses were conducted using the SAS Statistical Software version 9.4 (SAS Institute, Cary, NC) and R version 4.1.0 (2021-05-18).

## Results

We enrolled 512 Danish HTx recipients during the study period. Table 1 shows baseline characteristics of the recipients included. The median (IQR) age was 51 (38–58) and 393 recipients (77%) were males. The differences in age between the categories of multimorbidity and SEP were minor, except for cohabitation status and employment status (Supplementary Figure S2). We found no differences in the median number of multimorbidities between categories of SEP (Supplementary Table S4).

**TABLE 1:** Baseline characteristics of heart transplant recipients.

	Total
	**N = 512**
Gender	
Male	393 (77)
Female	119 (23)
Age	
Median (IQR)	51 (38–58)
Age groups	
0–20	50 (10)
21–40	93 (18)
41–60	301 (59)
+61	68 (13)
Follow-up time in years	
0–5	146 (29)
5–10	141 (28)
+10	225 (44)
Alive at end of follow-up	334 (65)
Cardiovascular morbidities (10 years prior to the index date)	
Myocardial infarction	175 (34)
Angina Pectoris	223 (44)
Heart failure	439 (86)
Heart valve diseases	59 (12)
Cardiac arrhythmias	245 (48)
Congenital heart disease	46 (9)
Cardiomyopathy	347 (68)
Cardiac inflammation	55 (11)
Aortic disease	NA
Peripheral arterial disease	33 (6)
Cerebrovascular disease	47 (9)
Cardiogenic shock and pulmonary edema	50 (10)
Hyperlipidemia	72 (14)
Other morbidities (10 years prior to the index date)	
Hypertension	62 (12)
Diabetes	59 (12)
Chronic obstructive pulmonary disease	58 (12)
Cancer	18 (4)
Chronic neurological disease	9 (2)
Chronic arthritis	NA
Chronic bowel disease	NA
Chronic liver disease	8 (2)
Chronic kidney disease	24 (5)
Chronic mental disease	NA
Mental disorder	NA
Multimorbidity (10 years prior to the index date)	
Number of chronic diseases, median (IQR)	1 (1–2)
Cardiovascular polypharmacy (180 days prior to the index date)	293 (57)
Cohabitation status	
Living alone	228 (45)
Cohabitation	284 (55)
Highest obtained educational degree	
Low (primary and lower secondary education)	165 (32)
Medium (upper secondary education and academy profession)	217 (42)
High (bachelor and above)	91 (18)
Not completed education (patients age ≤16 years)	28 (6)
Missing	11 (2)
Employment status	
Working	243 (48)
Not working	52 (10)
Early retirement	159 (31)
State pension	36 (7)
Under education	20 (4)
Missing	NA
Personal income group	
Low income (≤25th percentile)	103 (20)
Medium-high income (>25th percentile)	409 (80)

Values are n (%).

NA, not available (numbers less than five).

**FIGURE 1:**
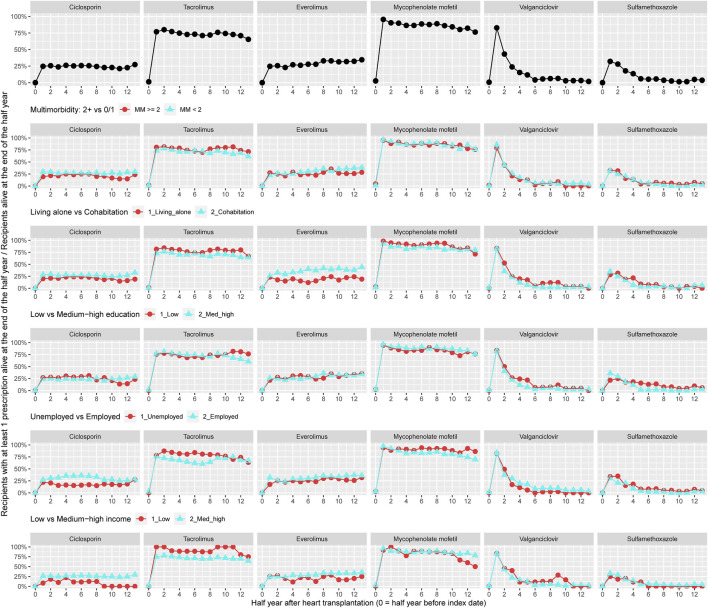
Prevalence of treatment with cost-free immunosuppression overall and by categories of multimorbidity and socioeconomic position.

Prevalence of treatment within the cost-free immunosuppressive regime is shown in Figure 1; though, only including HTx recipients (*n* = 258) recorded by the pharmacy at Aarhus University Hospital (Supplementary Table 6). During the 7 years follow-up, 25% of the recipients were on treatment with ciclosporin and the use of tacrolimus ranged between 68% and 82%. More than 95% used mycophenolate mofetil after heart transplantation and the prevalence decreased to 75% after 6 years. Recipients on treatment with everolimus steadily increased from 25% to 35%–36% within follow-up. Among recipients with at least two chronic diseases, a higher prevalence of treatment with tacrolimus was observed, whereas a lower prevalence of recipients used ciclosporin. A lower prevalence of treatment with everolimus was seen in recipients living alone. In recipients with low income, we observed a lower prevalence of use with ciclosporin and everolimus in contrast to higher prevalence of treatment with tacrolimus (Figure 1).

**FIGURE 2:**
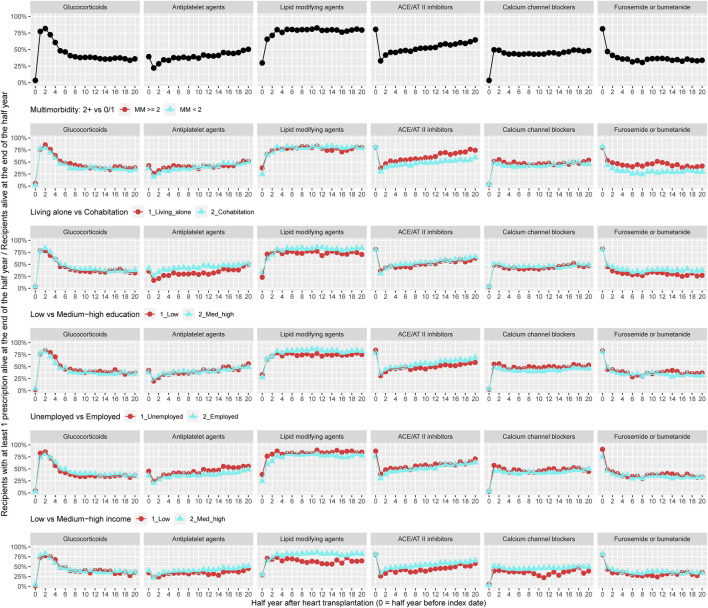
Prevalence of treatment with glucocorticoids and adjuvant pharmacotherapies overall and by categories of multimorbidity and socioeconomic position. ACE, Angiotensin-converting enzyme; AT, Angiotensin.

Figure 2 illustrates the prevalence of treatment with glucocorticoids and adjuvant pharmacotherapies within 10 years of follow-up for all recipients (*n* = 512). Prevalence of treatment with glucocorticoids decreased from 75% to 30% during follow-up. The prevalence of use with antiplatelet agents increased from 20% to 50% and treatment with lipid modifying agents was approximately 75% during follow-up. During the 10 years follow-up, prevalence of treatment with ACE/AT II inhibitors increased from 30% to 65%. Approximately 50% of recipients were in treatment with calcium channel blockers and 35% used furosemide or bumetanide (Figure 2). The prevalence of treatment with aldosterone antagonists and thiazides, respectively, was lower than 5% and not presented in Figure 2. We observed higher prevalence of use with antihypertensive medical therapies and loop diuretics in recipients with at least two chronic diseases. Among recipients living alone, prevalence of treatment with antiplatelet agents, lipid modifying agents, and furosemide or bumetanide during follow-up was lower. A lower prevalence of use of lipid modifying agents and ACE/AT II inhibitors was seen in recipients with low educational degree. Prevalence of treatment with lipid modifying agents, ACE/AT II inhibitors, and calcium channel blockers was lower among recipients with low income (Figure 2).

**FIGURE 3:**
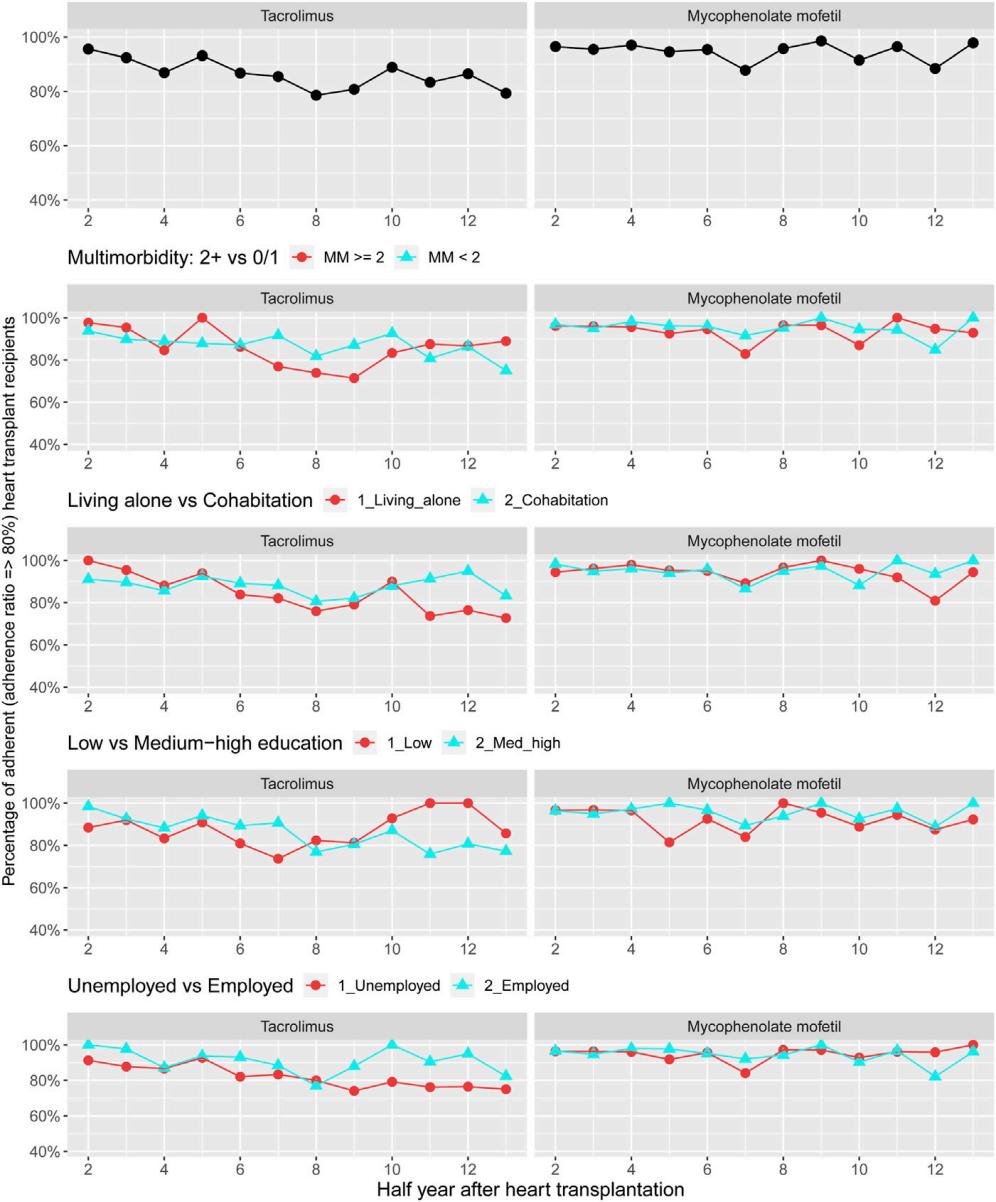
Prevalence of adherence to cost-free immunosuppressants overall and by categories of multimorbidity and socioeconomic position. Due to data protection, the variable of personal income was not included in the stratified illustrations.

Figure 3 shows the prevalence of adherence to cost-free immunosuppression therapy 1–7 years post-HTx. The overall prevalence for adherence was at least 80% for both treatment with tacrolimus or mycophenolate mofetil. Since less than 36% of the sub-recipients (*n* = 258) used ciclosporin or everolimus, we were not permitted as per IRB approval to present stratified adherence prevalence curves for these two medical therapies. We observed half-year periods with higher prevalence of non-adherence to both tacrolimus and mycophenolate mofetil in recipients with more than two chronic diseases. Half-year periods with higher prevalence of non-adherence to treatment with tacrolimus and mycophenolate mofetil, respectively, were seen in illustrations categorized by socioeconomic disadvantage; thus primarily observed for tacrolimus among recipients living alone, with a low educational degree, or unemployment (Figure 3). Due to data protection, variables for personal income were not included in the stratified illustrations.

**FIGURE 4:**
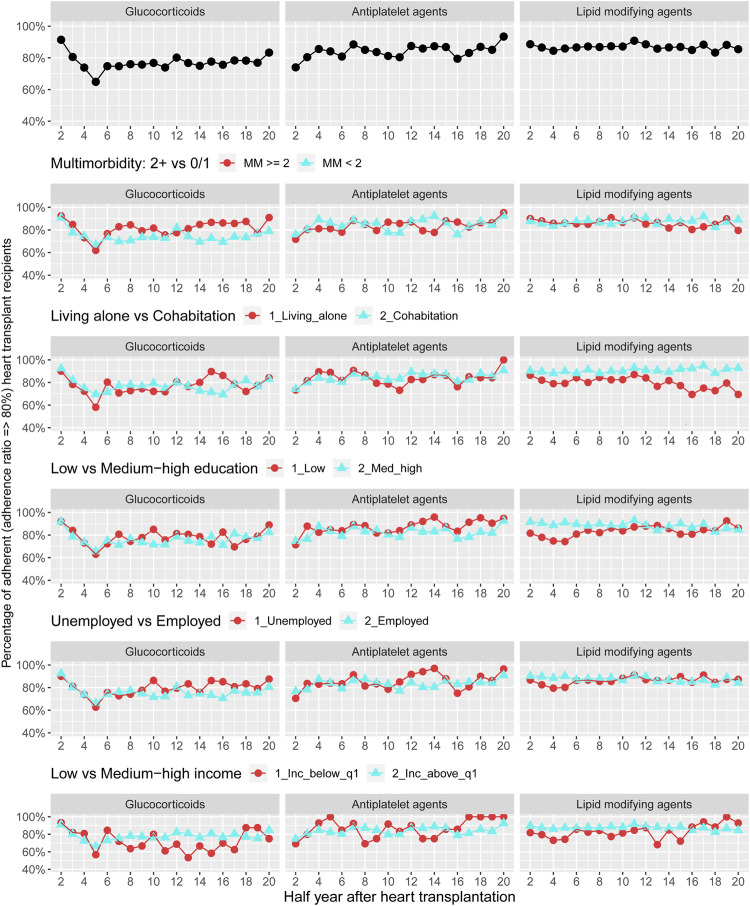
Prevalence of adherence to glucocorticoids and adjuvant pharmacotherapies overall and by stratified variables of multimorbidity and socioeconomic position.

**FIGURE 5:**
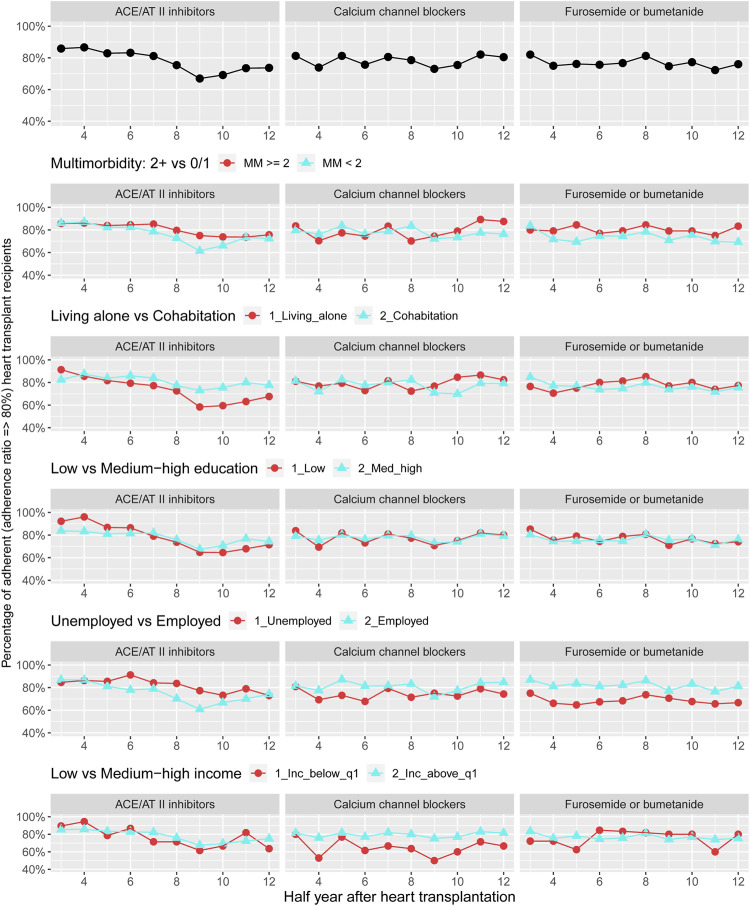
Prevalence of adherence to antihypertensive pharmacotherapies and loop diuretics overall and by categories of multimorbidity and socioeconomic position. ACE, Angiotensin-converting enzyme; AT, Angiotensin.

Figure 4 displays adherence curves regarding glucocorticoids and preventive pharmacotherapies 1–10 years after HTx. For glucocorticoids, we observed that the prevalence of adherence ranged between 65% and 92% during follow-up; prevalence of adherence to antiplatelet agents ranged between 75% and 95% and the prevalence of adherence to lipid modifying was approximately 85%–90%. We documented no pattern for adherence to glucocorticoids and preventive pharmacotherapies by multimorbidity. Among recipients with low income, we found half-year periods with higher prevalence of non-adherence to treatment with glucocorticoids. Half-year periods of higher prevalence of non-adherence were observed for lipid modifying agents in recipients living alone, with low educational level and low income (Figure 5) (Supplementary Figure S3).

Description of adherence to antihypertensive pharmacotherapies and loop diuretics 1–7 years after HTx are presented in Figure 5. We found that the overall prevalence of adherence to these medical therapies was 66%–88%. No pattern was observed in prevalence of adherence to antihypertensive pharmacotherapies and loop diuretics when categorized by multimorbidity. We observed that recipients living alone presented half-year periods of higher prevalence of non-adherence to ACE/AT II inhibitors. Half-year periods with a higher prevalence of non-adherence to calcium channel blockers and treatment with loop diuretics were seen in unemployed or low income group recipients (Figure 4) (Supplementary Figure S4).

## Discussion

This nationwide register study with longitudinal follow-up from 1995–2018 showed that in first-time HTx recipients with multimorbidity, the prevalence of treatment with antihypertensive pharmacotherapies and loop diuretics were higher. In socioeconomically disadvantaged recipients, both the number of patients treated with and adherence to cost-free everolimus, lipid modifying agents, ACE/AT II inhibitors, calcium channel blockers, and loop diuretics were lower. This was particularly pronounced in recipients living alone or with low income.

Multimorbidity is typically defined as the coexisting of two or more chronic conditions and has been shown to be associated to both the medical regime complexity as well as to non-adherence to the multiple medical therapies [30]. In accordance with our study, a small single-center study (*n* = 60) evaluating patient-level medication complexity over time showed that 5 years after surgery, HTx recipients were treated with increasing amounts of immunosuppressants, antihypertensives (81.8% used ACE/AT II inhibitors), and lipid modifying agents (98.3% used statins) to treat both existing and new-onset morbidities [7] as well as complications (allograft vasculopathy, graft failure, hypertension, cardiovascular diseases, and kidney disease, etc.) [3]. A smaller Spanish study [14] including adult chronic-stage (follow-up >1.5 years) HTx recipients (*n* = 135) demonstrated a relation between multimorbidity and worse patient-level Medication Regimen Complexity Index score (pMRCI). The pMRCI score was primarily influenced by the medical treatment of new-onset comorbidities [14]. This could indicate the need for strategies to reduce medication complexity and support self-management in long-term HTx survivors with increasing multimorbidity.

To our knowledge, this is the first study to describe register-verified (as opposed to self-reported) initiation of pharmacotherapies after HTx by individual-level SEP indicators in a universal healthcare system. We found that socioeconomic deprivation seems to influence lower initiation of cost-free treatment with everolimus, antiplatelet agents, lipid modifying agents, ACE/AT II inhibitors, calcium channel blockers, and loop diuretics; however, this mainly applied to recipients living alone or in the low-income group. We can only speculate how mechanisms of socioeconomic disadvantage in HTx recipients may affect initiation of pharmacotherapies. A nationwide population-based study among Danish patients with incident heart failure and reduced ejection fraction (*n* = 15.290) investigated the association between socioeconomic factors and quality of care (guideline-recommended process performance measures) [31]. The authors demonstrated that living alone, low-level education, and income in the lowest tertile were associated with reduced number of prescriptions redeemed for recommended medical therapies [31]. Thus, life-long complex pharmacological regimen and rigorous follow-up to monitor graft function and prevent new-onset comorbidities after HTx, may require well-coordinated multidisciplinary care, recipient engagement, and self-management [3], which could be negatively affected in socioeconomic disadvantaged patients. However, further studies are necessary to examine whether any interventions targeted against this socioeconomic imbalance can be of benefit.

Evidence is sparse regarding the association between multimorbidity and non-adherence to the life-long pharmalogical regime post-HTx. A study using meta-analytical methods reported an overall non-adherence rate of 14.5 cases per 100 recipients per year after HTx [10]. A review (2021) [21] documented that non-adherence to immunosuppressants in HTx recipients differed considerably (25%–40%), however, with adherence rates higher than 80% in several studies [21]. It should be noted that adherence is self-reported in most studies [10, 21]. Similarly, our study more accurately verified that adherence by pharmacy registrations of cost-free immunosuppressants was higher than 80%. Our sub-analysis among cost-free immunosuppression pharmacy data also implies that multimorbidity could impact periods of non-adherence to mainly tacrolimus and mycophenolate mofetil and not adjuvant pharmacotherapies.

In the international BRIGHT study [7] (*n* = 1,380), non-adherence to the pharmacological management regime post-HTx (1–5 years) has been reported to be 82.7% concerning immunosuppressive medical treatment and 76.1% to co-medical treatment (BASSIS scale). This self-reported non-adherence was significantly (*α* = 0.05) higher to co-medications than to immunosuppressants (adjusting for data clustering and center levels) [7]. Consistent with these studies, we observed periods of the lowest prevalence of adherence in adjuvant pharmacotherapies as ACE/AT II inhibitors (60%) during follow-up. In the present study, the documented register-verified description of higher prevalence of half-year periods of non-adherence to cost-free immunosuppressants compared with adjuvant pharmacotherapies when categorized by multimorbidity could be underpowered and results thus coincidental. Nonetheless, the observed differences in adherence between pharmacotherapies may be partly explained by differences in multimorbidity and the recipients expected efficacy versus side effects by multiple medical therapies. Thus, prioritizing of certain pharmacotherapies was reflected in recipients’ self-management behavior [30].

Socioeconomic inequality in adherence to pharmacotherapies after HTx has been demonstrated in four previous studies. A study from the United States [17] using the UNOS register (*n* = 33.893) showed that low neighborhood socioeconomic status (index score) was associated with higher risk of non-compliance to immunosuppressive treatment (HR 1.76) [17]. A second analysis of data from the international BRIGHT study [32] examined cost-related medication adherence (CRMNA) to immunosuppressive pharmacotherapies in recipients undergoing HTx. Self-reported items in 1,365 recipients measured CRMNA on average 3.35 ± 1.38 years after surgery. CRISMA was positively associated with being single (OR 2.29, 95% CI 1.17–4.47) and costs being a barrier (OR 2.60, 95% CI 1.66–4.07) [32]. In a single-center Chinese study (*n* = 168), adherence to immunosuppressants (BAASIS scale) showed that monthly income (<3,000 Chinese Yuan) correlated with non-adherence (OR 3.11, 95% CI 1.58–6.12) [33]. Our findings that mainly recipients living alone and those with low income have half-year periods of higher prevalence of non-adherence to treatment with tacrolimus, mycophenolate mofetil, glucocorticoids, lipid modifying agents, ACE/AT II inhibitors, calcium channel blockers, and loop diuretics agree with these four previous studies. We also found that recipients living alone were younger than those cohabiting. Age-based differences in non-adherence to medical therapies post-HTx were demonstrated in a single-center study from Germany (*n* = 858) [11]. The overall prevalence of adherence by the ITAS scale was 72.4% and positively associated with age (*p* < 0.001) [11]. Furthermore, a meta-analysis [34] assessed the impact of social support on organ transplant outcomes (including HTx). Married compared to unmarried recipients experienced 1.46 higher odds of adherence to pharmacotherapies [34]. Our findings are in line with previous studies showing register-verified measures of non-adherence to pharmacotherapies and individual-level indicators of economic and social disadvantage that could facilitate inequalities in self-management ability.

We have not identified other studies investigating the impact of educational level or employment on adherence to the life-long pharmacological management regime after HTx. Our study described that lower prevalence of initiation with ACE/AT II inhibitors and lipid modifying agents as well as half-year periods with higher prevalence of non-adherence to tacrolimus and lipid modifying agents were seen in recipients with low educational degree. Half-year periods with higher prevalence of non-adherence to tacrolimus, calcium channel blockers, and furosemide or bumetanide treatment were observed among unemployed recipients. Unemployed recipients in our study were approximately 3 years older than those employed, and the differences could thus be the result of confounding from age. However, our results could reflect inequalities in both pharmalogical treatment and self-management according to degree of education and employment status. This indicates the need for more focus on these individual indicators of socioeconomic deprivation also in countries with universal healthcare systems. In the view of this, a Danish study in heart-transplant recipients (*n* = 649) suggested that non-adherence to pharmacotherapies in socioeconomic disadvantages recipients seems to lead to a poorer prognosis [27]. During 1–10 years after HTx, low educational level (adjusted HR 1.66, 95% CI 1.14–2.43) and low income (adjusted HR 1.81, 95% CI 1.02–3.22) were associated with a first-time MACE (composite of readmission due to heart failure, graft failure, percutaneous coronary intervention, and all-cause mortality) [27].

Overall, our findings highlighted that, even in countries with free access to healthcare services and free- or low cost pharmacotherapy, integrated life-long adherence assessment to both immunosuppressive pharmacotherapies and adjuvant pharmacotherapies requires awareness. In the BRIGHT study, multidisciplinary teams, specified patient-centered practice, and higher degree of chronic illness management was associated with higher prevalence of adherence [12, 35, 36]. Interestingly, recent reviews [21, 37] indicate it could be helpful to electronically monitor long-term adherence by validated self-reported adherence questionnaires. We suggest paying attention to the organization and delivery of healthcare services also in universal healthcare systems, because some socioeconomically disadvantaged HTx recipients with multimorbidity may benefit from more individualized strategies to improve initiation and adherence to life-long pharmalogical management regime.

Data from Danish registers are validated for epidemiological research and have high completeness [22]. Using pharmacy records reduces potential recall bias, the risk of recipients changing behavior during observation, and “dose dumping” to appear more adherent. However, pharmacy records only account for pharmacotherapies dispensed and do not show if and how the medical therapies were used by recipients. We only included pharmacotherapies redeemed using at least one reimbursed prescription within 180 days. Thus, we cannot exclude potential misclassification of adherence to pharmacotherapies started late after transplant. Since the prescribed daily dose is not included in Danish Pharmacy records [28], surrogates for gold standards must be used. Consequently, the definition of adherence relies on assumptions and it may therefore be reasonable to assume that the variable used will involve some residual confounding caused by misclassification. We did not censor hospital stays as it is shown to have minor impact on PDC estimates [38]. We found no indication of any difference between the two Danish transplant centers and no indication of selection bias in the sub-analysis. Our inclusion of two centers working with very similar protocols and nationwide approach is a major strengths. The most pronounced limitation of the present study is the descriptive statistical approach due to small sample size; especially in the sub-study. As an example, description of the pharmacological regimes by decades is relevant due to changes in immunosuppressants and adjuvant medical treatment. However, the small number of recipients within the common time periods (1995–2001, 2002–2009, 2010–2019) makes the graphical illustrations too sensitive. Due to regulations, we are not allowed to present results stratified by decades [23]. Finally, we used independently collected individual-level information of multimorbidity and SEP of high validity only estimated at baseline.

In conclusion, our nationwide register study revealed that in first-time HTx recipients with multimorbidity, treatment with antihypertensive pharmacotherapies and loop diuretics were higher. Among socioeconomic disadvantages recipients, both treatment with and adherence to cost-free everolimus and adjuvant pharmacotherapies were lower.

## Data Availability

Data underlying this study cannot be shared publicly because of Danish legislation. Study data, statistical plan, and log-files can be made available through proposal to the Project Database (ID: 707738) at Statistics Denmark. Requests to access the datasets should be directed to https://www.dst.dk/en/TilSalg/Forskningsservice. Foreign researchers can access data from Statistics Denmark through an affiliation with a Danish-authorized research institution.

## Abbreviations

ATC, anatomical therapeutic chemical; CRS, civil registration system; DNPR, Danish national patient registry; HTx, heart transplantation; MACE, major adverse cardiovascular event; NPR, Danish national prescription registry; PCRR, psychiatric central research registry; SEP, socioeconomic position; STD, Scandiatransplant database.
