# Supplementary material for: Adherence to Pharmacotherapies After Heart Transplantation in Relation to Multimorbidity and Socioeconomic Position: A Nationwide Register-Based Study
Source: Transpl Int. 2023 Oct 11;36:11676. doi: 10.3389/ti.2023.11676 (PMC10599149; doi:10.3389/ti.2023.11676)
Supplement: Supplementary file 1 [file DataSheet1.PDF]

SUPPORTING INFORMATION

Adherence to Pharmacotherapies after Heart Transplantation in relation to Multimorbidity and Socioeconomic Position: A Nationwide Register-based Study

Contents

Table S1 International Classification of Diseases codes of algorithm to measure multimorbidity

Table S2 Classification of individual-level socioeconomic position

Table S3 Anatomical Therapeutic Chemical Classification codes of pharmacological management regime and Gold Standard Dose

Table S4 Multimorbidity by stratified socioeconomic position

Table S5 Baseline characteristic in heart transplant recipients, Aarhus Transplant Center

Figure S1 Calculation the Median Daily Dose (MDD)

Figure S2 Categories of multimorbidity and socioeconomic position by median (interquartile range)

Figure S3 Ratio of adherence to glucocorticoids and preventive pharmacotherapies overall and by categories of multimorbidity and socioeconomic position

Figure S4 Ratio of adherence to antihypertensive pharmacotherapies and loop diuretics overall and by categories of multimorbidity and socioeconomic position

Table S1 International Classification of Diseases codes of algorithm to measure multimorbidity [1-4]

**Included CDGs (grey) and included diseases (white)**	**ICD-8**	**ICD-10**
**Cardiovascular disease [5,6]**		
Myocardial infarction	410	I21
Angina Pectoris		
Unstable	411	I200
Stable	413	I20 (without I200), I251, I259
Heart Failure	42709, 42710, 42711, 42719, 42899,78249	I500, I501, I502, I503, I508, I509, I110, I130, I132, I420, I426, I427, I428, I429
Heart Vale disease		
Mitralvalve-insufficiency and stenosis	394	I05, I34, I390, I511A
Aorticvalve-insufficiency and stenosis	395	I06, I35, I391
Cardiac arrhythmia		
Atrial fibrillation or flutter	42793, 42794	I48
Bradycardia (sinus node dysfunction and AV-block)	42720, 42721, 42722, 42723	I440, I441, I442, I443, use 145 (*I455A, I455B, I455C, I455G)*
Ventricular tachycardia/fibrillation	42797, 42791	I470, I472, I490
Cardiac inflammation		
Endocarditis	421	I33, I38, I398
Myocarditis	422	I40, I41, I090, I514
Pericarditis	39109, 393, 420, 423	I30-132
Aortic disease		
Aortadissection	44109	I710
Aneurisme/dilatation	44110, 44111, 44119, 44120, 44121, 44129, 44199	I711-I716, I718-I719
Peripheral arterial disease	44389-44399	I739A
Cerebrovascular disease	430-438	I60-I69, G45, G46
Cardiogenic shock and pulmonary edema	427.10, 427.11	J81, I501B, R570
Congenital heart disease	746.00–747.49, 759.00, 759.01, 759.09	Q200-Q269, Q893
Cardiomyopathy	425	I42-I43 (excluding I42.6)
Hyperlipidemia (not included in the algorithm-only included as baseline characteristic)	*27200*	*E780*

**Hypertension**		
Hypertension	400-404	I10-I15
**Diabetes**		
Diabetes with end organ damage type1 type2	249.00, 249.06, 249.07, 249.09, 250.00, 250.06, 250.07, 250.09	E10-E14. O24 (except O24.4), G63.2, H36.0, N08.3
**Chronic obstructive pulmonary disease [5,6]**		
COPD	490-493; 515-518	J40–J47; J60–J67; J68.4; J70.1; J70.3; J84.1; J92.0; J96.1; J98.2; J98.3
**Cancer [5,6]**		
Cancer	140–209	C00-C97
**Chronic neurological disease [6]**		
Epilepsy	345	G40 (ex. G40.4), G41
Parkinson's disease	342	G20-G22
Multiple sclerosis	340	G35
**Chronic arthritis [6]**		
Rheumatoid arthritis/connective tissue disease	696.09, 712, 715	L40.5, M05-M07
**Inflammatory bowel disease/Chronic bowel disease [6]**		
Colitis ulcerosa and Mb. Crohn	563	K50-K51
**Chronic liver disease [6]**		
Chronic viral hepatitis,<br>Chronic liver disease	571-573	B18<br>K70-K76
**Chronic kidney disease [6]**		
Chronic pyelonephritis/Interstitial nephritis	590.09, 593.20	N11, N14, N15, N16
Other and unknown chronic renal disease (none of those above):	792, 584	N18-N19, N26, N27, N07, N08 (without N08.3)
**Chronic mental disease [5,6]**		
Schizophrenia	295.x9, 296.89,	F20 –F29
And related disorders/psychotic disorders	297.x9, 298.29-298.99, 299.04, 299.05, 299.09, 301.83	

Affective disorders incl. depression	296.x9 (excluding 296.89), 298.09, 298.19, 300.49, 301.19	F30-39
Dementia		
Alzheimer's disease	290.10	F00 (includes F00.0x, F00.1x, F00.2x, and F00.9x); G30 (includes G30, G30.0, 30.1, 30.8, 30.9)
Vascular dementia	293.09, 293.19	F01 (includes F01.0x, F01.1x, F01.2x, F01.3x, F01.8x, & F01.9x)
Other dementia	094.19 and 292.09; 290.09, 290.11, 290.18, 290.19, 292.09	F02; F03; F05.1; F1x.73 (F10.73 through F19.73); G23.1; G31.0A*, G31.0B*, G31.1, G31.8B, G31.8E
Other exclusions: Mild cognitive impairment (MCI) and amnestic syndromes. Applies when identifying cases and controls free from dementia. Not used to identify incident dementia	291.19	F04, F04.9, F05.1, F06.7 and F06.7x; F1x.6 (F10.6, F18.6, F19.6)
Neurotic, stress-related and somatoform disorder	300.x9 (excluding 300.49), 305.x9, 305.68, 307.99	F40-F48
Eating disorder	305.60, 306.50, 306.58, 306.59	F50

CDG = Comprehensive groups of Chronic Diseases; ICD = International Classification of Disease.

1. Schmidt M, Schmidt SA, Sandegaard JL, Ehrenstein V, Pedersen L, Sorensen HT. The Danish National Patient Registry: a review of content, data quality, and research potential. *Clinical epidemiology.* 2015;7:449-490.
2. Sundbøll J, Adelborg K, Munch T, et al. Positive predictive value of cardiovascular diagnoses in the Danish National Patient Registry: a validation study. *BMJ open.* 2016;6(11):e012832.
3. Adelborg K, Sundbøll J, Munch T, et al. Positive predictive value of cardiac examination, procedure and surgery codes in the Danish National Patient Registry: a population-based validation study. *BMJ open.* 2016;6(12):e012817.
4. Mors O, Perto GP, Mortensen PB. The Danish Psychiatric Central Research Register. *Scandinavian journal of public health.* 2011;39(7 Suppl):54-57.
5. Diederichs C, Berger K, Bartels DB. The measurement of multiple chronic diseases--a systematic review on existing multimorbidity indices. *The journals of gerontology Series A, Biological sciences and medical sciences.* 2011;66(3):301-311.
6. Barnett K, Mercer SW, Norbury M, Watt G, Wyke S, Guthrie B. Epidemiology of multimorbidity and implications for health care, research, and medical education: a cross-sectional study. *Lancet (London, England).* 2012;380(9836):37-43.

Table S2 Classification of individual-level socioeconomic position [1-5]

**Individual-level socioeconomic position**		**Dichotomized**
Cohabitation status	Alone (single, divorced, widow, etc.)<br>Cohabitating (married or in registered partnership)	**Alone**<br>**Cohabitating**<br>*(Recipients <18 years not included)*
Highest attained educational degree	Low (no formal education; primary and lower secondary education)<br>Medium (upper secondary education and academy profession degree)<br>High (bachelor and above)<br>Not completed education (patients under age of 16 years)<br>Missing	**Low education** (low degree)<br>**Medium-high education** (medium + high degree)<br><br>(*Recipients <18 years not included)*<br>*(Missing not included)*
Employment status	Working<br>Not working<br>Early retirement<br>State pension<br>Under education<br>Missing	**Unemployed** (not working, early retirement)<br>**Employed** (working, state pension, under education)<br><br><br>*(Recipients <18 years not included)*<br>*(Missing not included)*
Personal income	Based on the annually reported (since 1995) nationwide personal (pre-tax total) income statistics, we selected the 25th, 50th and 75th percentiles and then used the 25th percentile as a cut-off value for a binary income categorization: lowest (≤ 25th percentile) and medium-high (> 25th percentile).<br><br>Data on personal (pre-tax total) income were obtained from the Income Statistics Register [5]<br>The annual personal (pre-tax-total) income 25th, 50th and 75th percentiles in the National Danish Population was available from 1995, and thus income was estimated by linear regression from 1993-1995. They are based on the numbers downloaded from the STATBAK.DK at January 13, 2021.<br><br>https://www.statbank.dk/statbank5a/SelectTable/Omrade0.asp?SubjectCode=04&ShowNews=OFF&PLanguage=1	**Low income** (≤25th percentile)<br>**Medium-high income** (>25th percentile)<br><br>*(Recipients <18 years not included)*

1. Galobardes B, Shaw M, Lawlor DA, Lynch JW, Davey Smith G. Indicators of socioeconomic position (part 1). *Journal of epidemiology and community health.* 2006;60(1):7-12.
2. Schmidt M, Pedersen L, Sorensen HT. The Danish Civil Registration System as a tool in epidemiology. *European journal of epidemiology.* 2014;29(8):541-549.
3. Petersson F, Baadsgaard M, Thygesen LC. Danish registers on personal labour market affiliation. *Scandinavian journal of public health.* 2011;39(7 Suppl):95-98.
4. Jensen VM, Rasmussen AW. Danish Education Registers. *Scandinavian journal of public health.* 2011;39(7 Suppl):91-94.
5. Baadsgaard M, Quitzau J. Danish registers on personal income and transfer payments. *Scandinavian journal of public health.* 2011;39(7 Suppl):103-105.

Table S3 Anatomical Therapeutic Chemical Classification codes of pharmacological management regime and Gold Standard Dose [1]

**Medical therapies**		**ATC Classification**	**Gold standard for prescribed daily dose**
Cost-free Immunosuppression	Ciclosporine	L04AD01	75-100 mg x 2 daily
	Tacrolimus	L04AA06	1 mg x 2 daily<br>[prolonged-released capsules 1-3 mg x 1 daily]
	Everolimus	L04AD02	0.5–0.75 mg x 2 daily
	Mycophenolate mofetil	L04AA18	500 mg x 2 daily
	Valganciclovir	J05AB14	450 mg x 2 daily
	Sulfamethoxazole with trimethoprim	J01EE01	480 mg x 1 daily
Glucocorticoids	Glucocorticoids	H02AB	5-7.5 mg x 1 daily
Co-medical treatment	Antiplatelet agents	B01AC06, B01AC04	Clopidogrel 75 mg x 1 daily<br>Aspirin 75 mg x 1 daily
	Lipid modifying agents	MC10AA	Simvastatin, Pravastatin, Fluvastatin, Atorvastatin 40-80 mg x 1 daily<br><br>Lovastatin, Rosuvastatin 20 mg x 1 daily
	ACE/AT II inhibitors (Angiotensin converting enzyme inhibitors and Angiotensin II receptor antagonists)	C09A, C09B, C09C, C09D	Calculated the median daily dose (MDD)
	Aldosterone antagonists	C03DA01- C03DA04	Calculated the median daily dose (MDD)
	Calcium channel blockers	C08	Calculated the median daily dose (MDD)
	Thiazides	C03AA	Calculated the median daily dose (MDD)
	Furosemide or bumetanide	C03CA, C03EB	Calculated the median daily dose (MDD)

ACT = Anatomical Therapeutic Chemical Classification.

1. Pottegard A, Schmidt SAJ, Wallach-Kildemoes H, Sorensen HT, Hallas J, Schmidt M. Data Resource Profile: The Danish National Prescription Registry. *International journal of epidemiology.* 2017;46(3):798-798f.

Table S4 Multimorbidity by stratified socioeconomic position

Socioeconomic indicators	Number of chronic diseases, median (IQR)
Alone	1 (1-2)
Cohabitating	1 (1-2)
Low education	1 (1-2)
Medium-high education	1 (1-2)
Unemployed	1 (1-2)
Employed	1 (1-2)
Low income	1 (1-2)
Medium-high income	1 (1-2)

Table S5 Baseline characteristic in heart transplant recipients,

Aarhus Transplant Center

	**Total**
	**N = 258**
**Gender**	
Male	206 (50)
Female	52 (20)
**Age in years**	
0 - 20	26 (11)
21 - 40	43 (17)
41 - 60	158 (61)
+ 61	31 (12)
**Follow-up time in years**	
≤ 1 - 5	70 (27)
≤ 5 - 10	67 (26)
10 +	121 (47)
**Cardiovascular morbidities**	
Myocardial infarction	81 (31)
Angina Pectoris	
Heart failure	215 (83)
Heart valve diseases	27 (11)
Cardiac arrhythmias	132 (51)
Congenital heart disease	25 (10)
Cardiomyopathy	171 (66)

Cardiac inflammation	22 (9)
Aortic disease	NA
Peripheral arterial disease	20 (8)
Cerebrovascular disease	18 (7)
Cardiogenic shock and pulmonary edema	13 (5)
Hyperlipidemia	38 (15)
**Other morbidities**	
Hypertension	29 (11)
Diabetes	34 (13)
Chronic obstructive pulmonary disease	29 (11)
Cancer	8 (3)
Chronic neurological disease	NA
Chronic arthritis	NA
Chronic bowel disease	NA
Chronic liver disease	6 (2)
Chronic kidney disease	11 (4)
Chronic mental disease	NA
**Multimorbidity (10 years prior to the index date)**	
Number of chronic diseases, median (IQR)	1 (1-2)
**Cardiovascular polypharmacy (180 days prior to the index date)**	165 (64)
**Cohabitation status**	
Living alone	114 (44)
Cohabitation	144 (56)
**Highest obtained educational degree**	
Low (primary and lower secondary education)	86 (33)
Medium (upper secondary education and academy profession)	114 (44)

High (bachelor and above)	42 (16)
Not completed education (patients age ≤16 years)	14 (5)
Missing	NA
**Employment status**	
Working	120 (47)
Not working	32 (12)
Early retirement	83 (32)
State pension	14 (5)
Under education	9 (4)
Missing	NA
**Personal income group**	
Low income (≤ 25th percentile)	48 (19)
Medium-high income (>25th percentile)	210 (81)

Values are n (%).

[a] Data available since 1995 in the Danish National Prescription Registry.

Figure S1 Calculation the Median Daily Dose (MDD)

Heart Transplantation Index date	1-Year		2-Year		3-Year		4-Year		5-Year		6-Year	
	**Days**		**Days**		**Days**		**Days**		**Days**		**Days**	
	0-180	181-360	361-540	541-720	721-900	901-1080	1081-1260	1261-1440	1441-1620	1621-1800	1801-1980	981-2160
	**Interval**	**Interval**	**Interval**	**Interval**	**Interval**	**Interval**	**Interval**	**Interval**	**Interval**	**Interval**	**Interval**	**Interval**
	1	2	3	4	5	6	7	8	9	10	11	12
			**Adherence (PDC) estimated by MDD-1**					**Adherence (PDC) estimated by MDD-2**				
	Blanking Period	Calculation MDD-1 by all prescriptions in the 2nd 180-day interval after index date					Calculation MDD-1 by all prescriptions in the 2nd 180-day interval after index date					

Figure S2 Categories of multimorbidity and socioeconomic position by median (interquartile range)

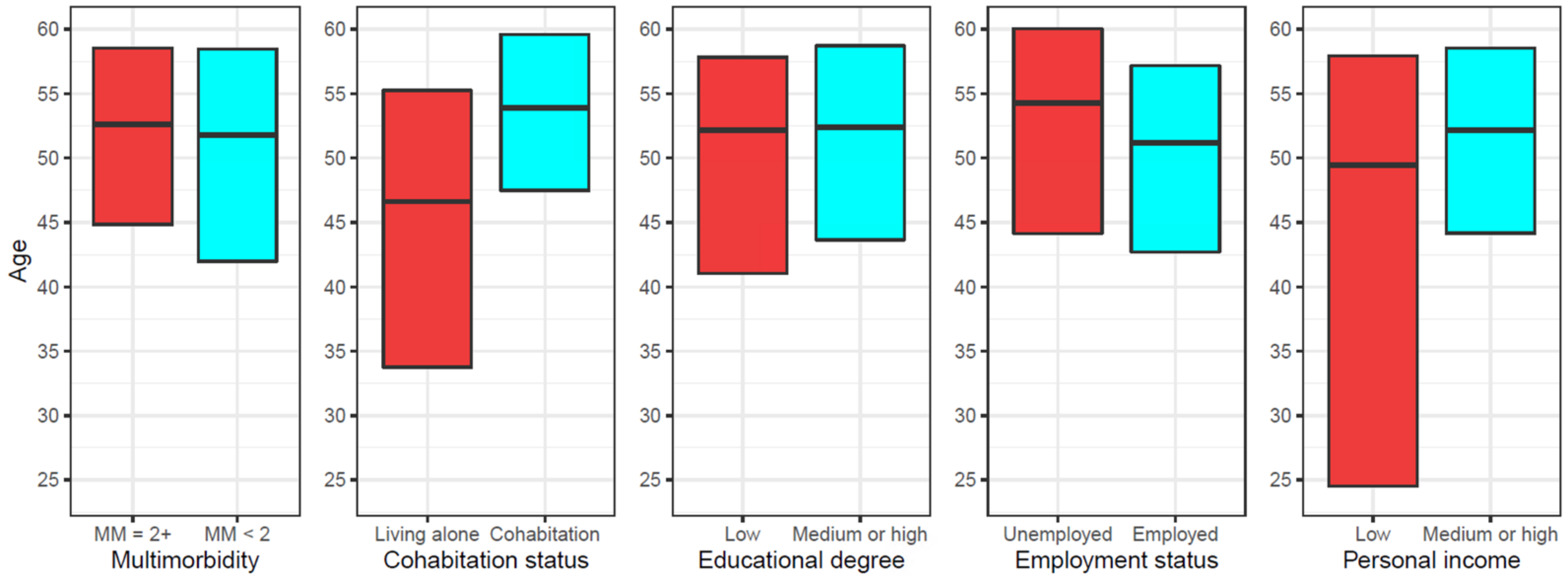

Figure S3 Ratio of adherence to glucocorticoids and preventive pharmacotherapies overall and by categories of multimorbidity and socioeconomic position

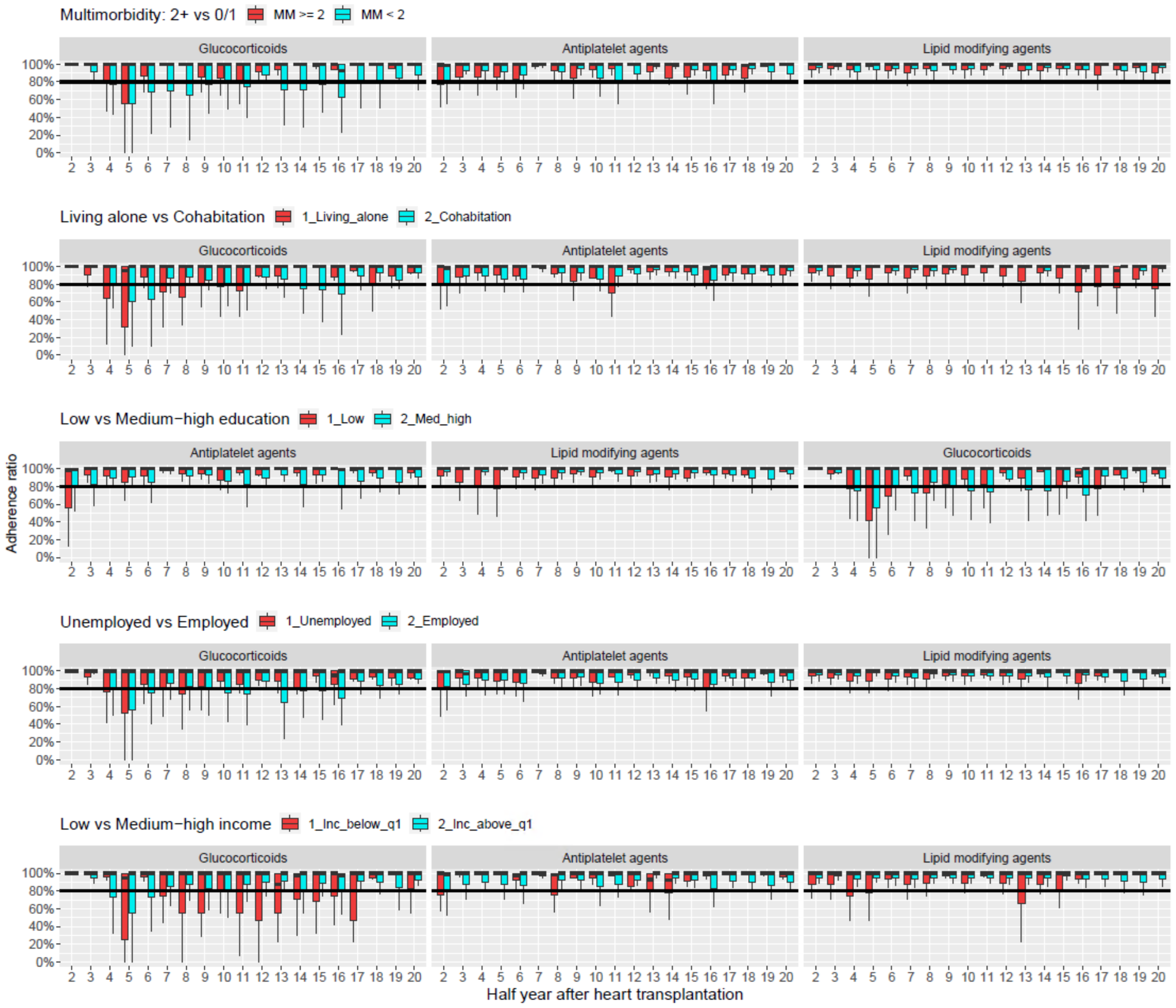

Figure S4 Ratio of adherence to antihypertensive pharmacotherapies and loop diuretics overall and by categories of multimorbidity and socioeconomic position

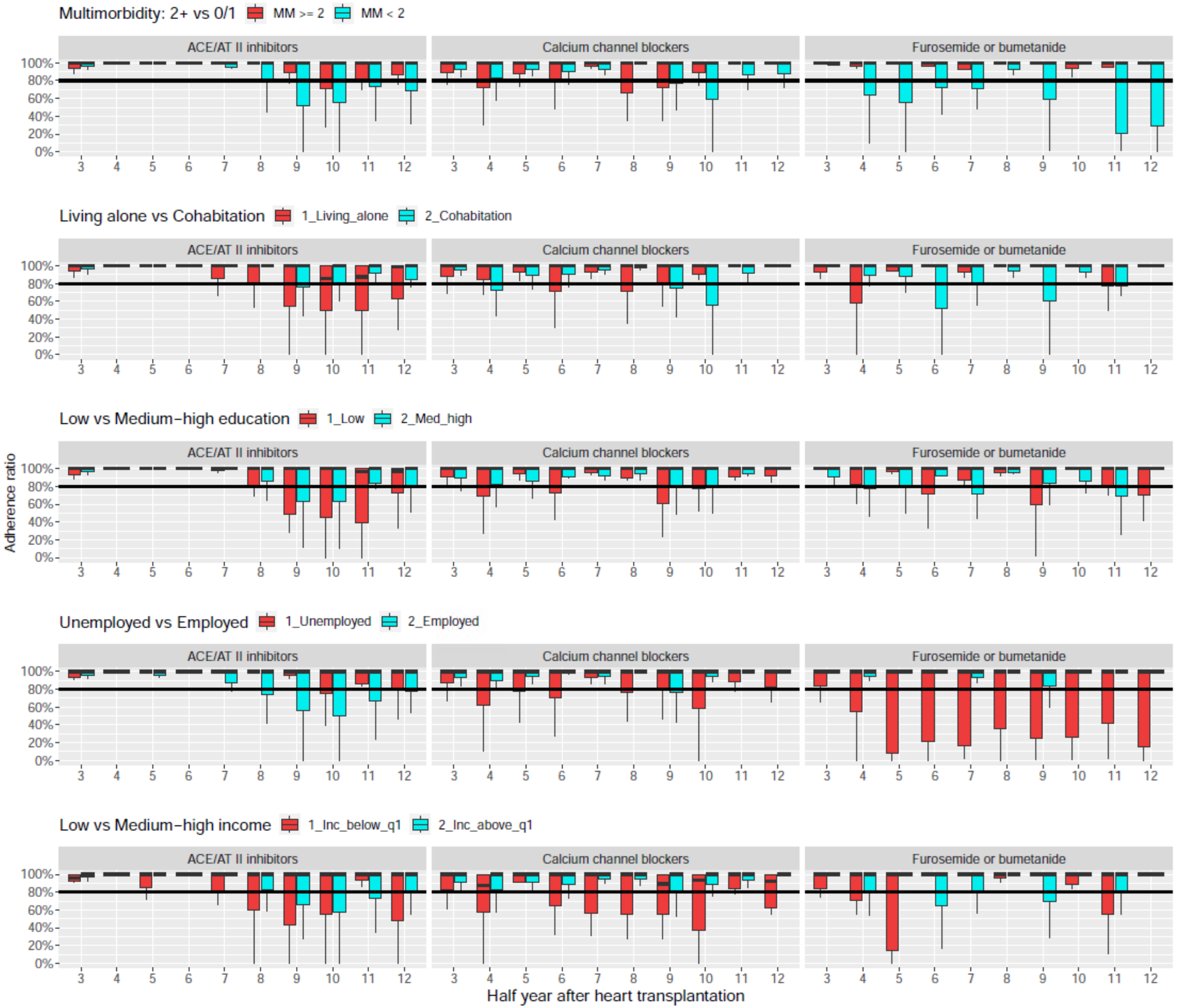

ACE, Angiotensin-converting enzyme
AT, Angiotensin
